# Self Management Activation Randomised Trial for Prostatitis (SMART-P): study protocol for a randomised controlled trial

**DOI:** 10.1186/1745-6215-12-210

**Published:** 2011-09-26

**Authors:** Mark Rochester, James Armitage, Mark Sanders, Paula Christmas

**Affiliations:** 1 Norfolk and Norwich University Hospital NHS Foundation Trust Colney Lane Norwich, NR4 7UY, UK; 2 Pain Management Centre Norfolk and Norwich University Hospital NHS Foundation Trust Norwich Community Hospital Site Bowthorpe Road Norwich, NR2 3TU, UK

## Abstract

**Background:**

Chronic prostatitis otherwise known as chronic pelvic pain syndrome is a common urological diagnosis that causes many men significant morbidity and has a detrimental effect on their quality of life. Standard treatment with antibiotics and simple analgesia are often ineffective and many patients are managed by the chronic pain services.

Cognitive behavioural therapy has been shown to be helpful in the management of many chronic diseases and has recently been proposed as an effective treatment for chronic prostatitis. Furthermore, a self management programme administered to groups of men with lower urinary tract symptoms has been shown to be more effective than standard treatments including surgery.

Therefore, we have developed a cognitive behavioural therapy programme specifically for men with chronic prostatitis. This novel treatment approach will be compared to conventional therapy in the pain clinic such as atypical analgesia and local anaesthetic injections in the context of a randomised controlled trial.

**Methods/Design:**

Men will be recruited from general urology outpatient clinics following the exclusion of other diagnoses that could be responsible for their symptoms. Men will be randomised to attend either a self management healthcare and education programme or to pain clinic referral alone. The self management programme will be administered by a clinical psychologist to small groups of men over six consecutive weekly sessions each lasting two hours. Patients will be taught techniques of problem-solving and goal-setting and will learn coping mechanisms and how to modify catastrophic cognition.

The primary outcome will be change from baseline in the National Institute of Health Chronic Prostatitis Symptom Index, a validated instrument for the assessment of men with chronic prostatitis. Secondary outcomes include generic quality of life scores and analgesic and drug usage. Outcomes will be assessed at 2, 6 and 12 months.

**Discussion:**

If this group administered self management programme is shown to be effective in the treatment of men with chronic prostatitis it may become the new standard of care for these patients. Furthermore, it may be adapted for use in women with interstitial cystitis, a condition which is analogous to chronic prostatitis in men.

**Trial Registration:**

Current Controlled Trials ISRCTN21012555

## Background

Prostatitis is a common urological diagnosis in men of all ages, representing 8% of male urology office visits [1]. Type III prostatitis, also referred to as chronic prostatitis/chronic pelvic pain syndrome (CP/CPPS) is the most common type and accounts for 90-95% of prostatitis diagnoses [2].

Standard care for men with CP/CPPS has traditionally consisted of initial assessment by a urological surgeon to exclude an underlying reversible organic cause for pain, followed by a non-uniform pathway that incorporates a variety of pharmacological approaches including antibiotics, non-steroidal anti-inflammatory drugs, alpha-adrenoceptor blockers, 5-alpha-reductase inhibitors and specialist pain-clinic approaches such as the use of gabapentin. That no one therapeutic approach is consistently beneficial underpins the fact that the underlying condition is multifactorial in its pathogenesis and includes physical, emotional and psychological components [3]. Effective treatment, therefore, must focus not only on the physical pathology but also the emotional and psychological aspects of the disorder. Psychosocial factors were recently targeted in the development of a cognitive-behavioural programme designed specifically for men with CP/CPPS [4]. Furthermore, a recent analysis of the large National Institute of Health Chronic Prostatitis Cohort showed that psychological variables could predict pain experience [5].

Self management interventions that enhance patients' problem solving and goal-setting skills have been shown to be effective for a number of chronic diseases including arthritis, diabetes and asthma through reduction in secondary care referrals, reduced primary care attendance and increased self efficacy [6]. More recently a group-directed self management programme has been shown to improve both symptoms and quality of life in men with lower urinary tract symptoms attributed to benign prostatic hyperplasia [7].

We have developed a programme of cognitive behavioural therapy for men with CP/CPPS. This intervention uses personal health planning and self directed care delivered in the context of small group sessions. We believe that through this holistic approach we can improve these patients' quality of life and functional status, reduce their pain and reduce the need for sustained primary and secondary care consultation.

The main objective of the study is to show that our programme of group-directed cognitive behavioural therapy for men with CP/CPPS is more effective than standard therapy (pain relief and coping mechanisms delivered in the pain clinic).

## Methods/Design

### Study Design

We believe that our self management programme would be best evaluated in the context of a randomised controlled trial compared to standard care. A randomised controlled trial is the most rigorous trial design that will allow comparison of the effectiveness of our intervention with standard treatment and will also allow an economic evaluation.

### Setting

The trial will take place at the Norfolk and Norwich University Hospital NHS Foundation Trust. The treatment sessions will take place at the Pain Management Centre and follow-up visits will be scheduled in the hospital outpatient department.

### Patients

We will recruit men with CP/CPPS that has not responded to simple treatment with painkillers or antibiotics from general urology outpatient clinics at Norfolk and Norwich University Hospital NHS Foundation Trust. All patients aged 18 or over with CP/CPPS referred for the first time by their GP will be eligible for inclusion. All patients will be assessed by a consultant urologist to diagnose CP/CPPS and exclude other important causes for their symptoms (for example, ureteric stones, prostate cancer).

Patients meeting these inclusion criteria will be offered the opportunity to take part in the study. Those who do not wish to take part will be offered standard care and be referred to the pain clinic if they wish.

If during the course of the study it becomes clear that further psychological treatment would be required for an unexpected diagnosis in a given participant, this would be made available.

Vulnerable patients, those who are unable to consent for themselves and patients who do not speak English will be excluded from the study. A detailed and explicit list of inclusion and exclusion criteria is provided in Table 1.

**Table 1:** Inclusion and Exclusion Criteria

*Inclusion Criteria*
• Men
• Age 18 years or order
• Diagnosis of Type III chronic prostatitis/chronic pelvic pain syndrome by a consultant urologist
• Failure of simple analgesia and antibiotics to control symptoms
*Exclusion Criteria*
• Prostate cancer or suspected prostate cancer
• Renal tract calculi
• Acute bacterial prostatitis
• Other urological and non-urological diagnoses that could account for a patient's symptoms
• Inability to comprehend and converse in the English language
• Vulnerable adults

A comprehensive list of the study inclusion and exclusion criteria.

### Power Calculation

**Figure 1:**
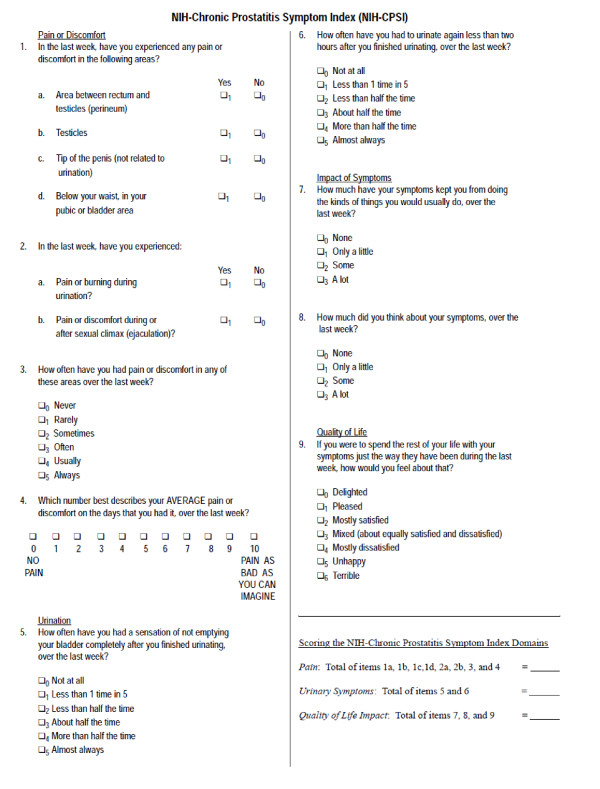
**NIH-Chronic Prostatitis Symptom Index**. The National Institutes of Health Chronic Prostatitis Symptom Index, a validated questionnaire for the assessment of men with CP/CPPS. It evaluates pain and urinary symptoms, and assesses the impact of the condition on function and quality of life.

This is based on the primary outcome measure, the National Institute of Health Chronic Prostatitis Symptom Index (NIH-CPSI, see Figure 1). Mean NIH-CPSI scores in published trials are around 25, with standard deviation in the region 6-7. To detect a mean fall in NIH-CPSI of 4 points, we used CPSI score in control of 25.2, and CPSI score of 21.2 in the intervention with standard deviations of 6.1 and 7.2 in the control and intervention respectively. At 90% power, 5% two sided significance level, we estimate a sample size of 59 individuals in each group. Taking account of 15% loss to follow-up, we will require 60 individuals in each group giving a total sample size of 120.

### Randomisation methods

Randomisation will be carried out by a method of Random Permuted Blocks so as to ensure exact equal treatment numbers at certain equally spaced time points in the sequence of patient assignments. The randomisation sequence will be generated using statistical software in random blocks, size of four individuals per block.

### Assessment of outcome

The primary outcome will be change from baseline in the NIH-CPSI, a validated instrument used for the assessment of men with CP/CPPS (see Figure 1) [8]. The NIH-CPSI assesses domains of pain, urinary function, sexual function, and the impact of symptoms on quality of life.

Secondary outcomes will include the SF-36, HAD (Hospital Anxiety and Depression) scale and PAM (Patient Activation Measurement) tool. The SF-36 will provide a measure of generic quality of life. The HAD scale is a self-screening questionnaire for depression and anxiety. The PAM tool will provide a measurement of empowerment and be used to evaluate the degree to which patients feel that they are in control of their health status. Changes in analgesic and drug requirement will be a secondary effectiveness outcome. An economic evaluation will also be undertaken as a secondary outcome measure.

### Intervention

The intervention comprises a total of six consecutive once-weekly cognitive behavioural therapy sessions delivered to small groups (5-8) of men with CP/CPPS. Each session will last 2 hours and will be led by a Consultant Clinical Psychologist with support from a Specialist Pain Nurse. There will be further input from a Consultant Pain Specialist and Senior Urologist if required particularly in the initial sessions. The sessions will focus on re-enablement through patient education, identifying and challenging negative thoughts, fostering a sense of control and introducing problem-solving and goal-setting strategies. The content of each session is briefly detailed in Table 2.

**Table 2:** Group-directed CBT health improvement intervention

	Content	Aim
*Week 1*	Introduction to programme. Ice-breaking exercise Information on CPPS (Consultant Pain Specialist) Set goals	Facilitate group to bond. Validates pain experience Promote re-engagement with valued activities
*Week 2*	Education re Gate Control Theory Identify gate openers Review goals Homework re goals Homework re gate closers	Validate pain experience Foster a sense of some control Increase valued activities/quality of life
*Week 3*	Education re Hot Cross Bun (CBT) Introduce Thought Records Homework: complete Thought Records	Introduce cognitive behavioural Principles Begin to identify automatic thoughts
*Week 4*	Thought Records Homework: complete Thought Records	Identify automatic thoughts and unhelpful behavioural response. Link to emotional state
*Week 5*	Challenging Thoughts Homework: Thought Challenging	Challenge unhelpful thoughts. Identify more appropriate thoughts Problem-solving strategies
*Week 6*	Communication Skills	Problems solving strategies Identify and reduce solicitous behaviour

A programme of group-directed cognitive behavioural therapy designed  specifically for men with CP/CPPS.

All patients randomised to the intervention arm will be able to leave the study at any time and all will be offered standard pain clinic care thereafter. We will carry out both intention to treat and per protocol analysis.

### Data Collection, Management and Analysis

Data will be collected and managed by a Specialist Research Nurse. The Specialist Research Nurse will be responsible for the day-to-day running of the trial as well as the collection of all administrative, demographic and outcome data. She will be the key contact for participants requiring support or advice throughout the study.

Outcome data will be collected at the time of enrolment into the study and again at 2, 6 and 12 months reviews after the commencement of treatment as part of a routine outpatient pain clinic review. The Specialist Research Nurse responsible for collecting these data will not be aware which treatment each patient has received.

### Statistical analysis

First descriptive and demographic statistics will be presented at baseline and follow-up measurements including measures of locations, for example mean or median and measures of spread including standard deviations or interquartile range depending on the distribution of measurements. Further, percentages and the 95% confidence intervals will be given for categorical data stratified by treatment group. All statistical tests will be carried out using two-sided tests at 5% significance level.

Given the repeated nature of our study, we will use the analysis of covariance approach recommended by Frison and Pocock for which the follow-up measurements are analysed at each follow-up time point adjusting for baseline and other confounding factors [9].

### Study Administration and Ethical Issues

Access to patient data will be restricted to the Specialist Research Nurse and all members of the NNUH CP/CPPS Research Group (see Table 3). All data will be anonymised. Paper records will be stored in locked filing cabinets and electronic data will be held on password-protected NHS computers.

**Table 3:** NNUH CP/CPPS Research Group

Mr Mark Rochester MA MB BChir MD MRCS(Eng) FRCS(Urol) Consultant Urological Surgeon Norfolk and Norwich University Hospitals NHS Foundation Trust Colney Lane Norwich, NR4 7UY

Mr James Armitage MBBS BSc(Hons) MD MRCS(Eng) FRCS(Urol) Specialist Registrar in Urology Norfolk and Norwich University Hospitals NHS Foundation Trust Colney Lane Norwich, NR4 7UY

Dr Mark Sanders MD FRCA Consultant Anaesthetist Norfolk and Norwich University Hospitals NHS Foundation Trust Colney Lane Norwich, NR4 7UY

Dr Paula Christmas Consultant Clinical Psychologist Pain Management Centre Norfolk and Norwich University Hospitals NHS Foundation Trust Norwich Community Hospital Site Bowthorpe Road Norwich, NR2 3TU

A list of all members of the Norfolk and Norwich University Hospitals  CP/CPPS Research Group.

The Public and Patient Involvement in Research (PPIRes) department at NHS Norfolk has been consulted in the development of this trial. Furthermore, a patient with CP/CPPS has been invited to steering group meetings and has been involved with the study design and development.

The Norfolk and Norwich University Hospital NHS Foundation Trust will be the lead sponsor for this study. Funding for the trial has been obtained through the NHS East of England Regional Innovation Fund.

The NHS Indemnity Scheme will meet the potential legal liability of the Norfolk and Norwich University Hospital NHS Foundation Trust for harm to participants in the conduct of the study.

### Resource Requirements

This study will require additional resources from the Norfolk and Norwich University Hospital NHS Foundation Trust. These include the involvement of a number of health care professionals in the provision of the healthcare intervention. A Specialist Research Nurse will also be required to co-ordinate the study and to collect data on effectiveness outcomes. Funding will be required for room hire and refreshments for the participants. Administrative expenses and licence fees for the validated instruments to assess outcomes constitute further costs.

These costs will be met entirely through funding from the NHS East of England Regional Innovation Fund (see Table 4).

**Table 4:** Breakdown of study costs

**Staff Involvement**
Urology Research Nurse, Band 6(30), 1.0WTE £42,551
Pain Specialist Nurse, Band 6(30), 0.4WTE £17,021
CBT-trained Clinical Psychologist, Band 8C, 0.2WTE £16,706
Admin Assistant, Band 3(12) 0.4WTE £9,246
Medical Statistician, Band 7 - based on PM, 5% £2,499
Consultant Input, 120 hours - 1 hour per participant £111.14 £13,337
SpR input - 120 hours, 1 hour per participant £39.65 £4,758

**Departmental Costs**
Room Hire for therapy sessions - 2 hourly sessions, 10 individual groups, 1 session per week for 8 weeks per group. (60 participants) £15.91per hour (£2,546)
Refreshments for group sessions (60 participants) £40.00 per patient (£2,400)
Research Assessment Room Hire: 4 appointments × 45 mins for 120 participants £15.91 per hour (£5,728)

**Direct Patient Costs**
Consumable costs for study - stationary, postage, telephone £20.00 per patient (£2,400)

**Other Costs**
R&D Sponsorship Costs £1,875
SF-36 and HADS Questionnaire Licence costs £3,200
ISR CTN Registration £200

**TOTAL RESEARCH COSTS** **£124,466**

A comprehensive listing of all of the research costs associated with the  SMART-P randomised controlled trial.

### Study Plan

Week 1:

Initial visit, enrolment, consent, baseline NIH-CPSI/SF36/HAD/PAM

Pain Management Centre.

Duration - approximately 20 minutes

Weeks 2:-7:

Therapeutic intervention as in Table 2

Pain Management Centre

Duration - approximately 120 minutes per session

Also at the conclusion of week 7, NIH-CPSI/SF36/HAD/PAM

Week 9-10:

Telephone follow-up with key contact to maintain engagement in both study arms and address concerns that may arise.

Week 31:

Six month follow-up assessment, NIH-CPSI/SF36/HAD/PAM

Pain Management Centre

Duration - approximately 20 minutes (Option of telephone or email follow-up depending on patient preference)

Week 33-34:

Telephone follow-up with key contact to maintain engagement in both study arms and address concerns.

Week 57:

Final follow-up assessment, NIH-CPSI/SF36/HAD/PAM

Pain Management Centre

Duration - approximately 20 minutes

**Table:** 

	Month															
	1	2	3	4	5	6	7	8	9	10	11	12	13	14	15	16

Ethics Approval																

Establish steering committee																

Recruit new staff																

First consultations with patients begin																

Group therapy sessions commence																

Recruiting 10-12 patients per month																

Review first patients' progress																

Initial data analysis																

Presentation of data at urology meeting																

### Dissemination and Outcome

All study participants will be contacted in writing to thank them for participating and to provide them with either a brief synopsis of the study findings and the implications for future practice or a full report depending on participant preference.

We intend to present the findings of the study at national and international urological meetings (for example, the annual meetings of the British, European and American Urological Asssociations). We also plan to publish the study findings in high impact peer reviewed medical journals.

We believe that the group-directed self management programme will be a more effective treatment for men with CP/CPPS than standard treatment. If this is demonstrated to be the case in this study it will have profound implications for the future care of this group of patients. We anticipate that group-directed self management may then become the new international standard of care for patients with CP/CPPS.

## Competing interests

The authors declare that they have no competing interests.

## Authors' contributions

MR and JA conceived the study, obtained funding and wrote the study protocol. PC and MS were responsible for developing the cognitive behavioural therapy sessions. All authors contributed to the study design and approved the final version of the manuscript for publication.
